# New insight into the global record of the Ediacaran tubular morphotype: a common solution to early multicellularity

**DOI:** 10.1098/rsos.231313

**Published:** 2024-03-20

**Authors:** Rachel L. Surprenant, Mary L. Droser

**Affiliations:** ^1^ Department of Earth and Planetary Sciences, University of California, Riverside, CA 92521, USA

**Keywords:** Ediacaran, database, tube, tubular organisms

## Abstract

The tubular morphogroup is a common component of Earth’s first complex, multicellular communities—the Ediacaran biota—and offers valuable insight into biological traits that are fundamental to animal life because they have intriguing links to metazoan phyla and are highly abundant in Ediacaran ecosystems. Biomineral tubes (e.g. *Cloudina*) are well described from the Nama assemblage (~550–538 Myr), yielding a relatively detailed understanding of this subset of the morphogroup. Conversely, the non-biomineral tubular taxa of the Nama assemblage, as well as of the older White Sea assemblage (~560–550 Myr), are poorly understood. As a result, the variability of characters that define non-biomineral tubular organisms is unknown and their diversity dynamics throughout the terminal Ediacaran are unconstrained. To test hypotheses related to the diversity, morphological variability and temporal distribution of non-biomineral tubes, a comprehensive database of non-biomineral Ediacaran tubular taxa was compiled. Results demonstrate previously unrecognized morphological disparity in the non-biomineral tubular morphogroup and reveal that it comprises a higher number of genera than all other non-tubular morphogroups in the White Sea and the Nama. Thus, it illustrates that a tubular form dominated Ediacaran ecosystems for considerably longer than previously appreciated and, importantly, was the most common solution to early multicellularity.

## 1. Introduction

The Ediacaran biota represent the oldest record of complex, community-forming animals and have traditionally been divided into three temporally distinct assemblages, including the Avalon (575–560 Myr), the White Sea (560–550 Myr) and the Nama (550–538 Myr) [1] (but see [2]). The Ediacaran tubular morphogroup, a suite of organisms characterized by their shared hollow and elongated morphologies, is recognized as a significant component of the Ediacara biota, associated with the White Sea and Nama assemblages, owing to their global distribution [3–5], high abundance [6–10] and links to metazoan phyla, including Cnidaria, Porifera and Annelida [7,11–15].

The common occurrence of a tubular form in the Ediacaran suggests that there was an advantage to this constructional morphology, the nature of which remains unknown. This is further supported by the fact that the tubular morphogroup is one of only four Ediacaran morphogroups (i.e. Arboreomorpha, Erniettomorpha, Rangeomorpha and tubular organisms) that occur in both the White Sea assemblage and the Nama assemblage. Not only does this demonstrate that tubes were key components of Ediacaran ecosystems but also that the morphogroup persisted across the White Sea–Nama extinction event, which is associated with a loss of 80% of Ediacaran genera and the extinction of four morphogroups [16]. Subsequent to the extinction event, the relatively depauperate communities of the Nama assemblage are dominated by an abundant tubular fauna thought to have radiated in and to be uniquely characteristic of the Nama assemblage [2,17–19].

The observed radiation of tubular organisms in the Nama assemblage is a well-documented event that has been demonstrated through a series of thorough analyses drawing primarily from the record of biomineral tubular taxa (e.g. [4,13]). The number of biomineral tubular genera, their characteristics and their temporal distributions have been well documented, with particularly thorough insight being provided by Selly *et al*. [4, table 1, fig. 10] and by Yang *et al*. [13, figs 5 and 7]. Comparatively little is known about the non-biomineral members of the tubular morphogroup which first appear in the White Sea assemblage and persist into the Nama assemblage. Despite reports of abundant non-biomineral tubes from the Nama assemblage [2] and the White Sea assemblage [5,10,15,20–27], in some cases occurring in such high abundance that they shaped ecosystems by limiting available ecospace [28–30], there is no comprehensive understanding of the number of genera that comprise the non-biomineral subset of the tubular morphogroup, their morphological characteristics or temporal variability. As a result, the radiation of tubes in the Nama assemblage remains uncontextualized by the diversity of the tubular taxa that preceded them, obfuscating the scale and nature of tubular diversification in the terminal Ediacaran.

This study addresses these outstanding questions through the creation and analysis of a database containing all occurrences of non-biomineral Ediacaran tubular organisms. The database is the first comprehensive global study of non-biomineral Ediacaran tubular organisms and is used to accomplish two primary goals geared towards providing novel insight into the non-biomineral tubular morphogroup: (i) identify the number of non-biomineral tubular genera and define the extent of their morphological variability, including gross morphology and external characters and (ii) describe the temporal variability in the number of genera and the constructional complexity of non-biomineral tubular organisms in the terminal Ediacaran.

Results demonstrate a previously unrecognized diversity of gross morphologies and external characteristics in the non-biomineral tubular morphogroup, which is contrary to their common description as exceedingly simple organisms. Additionally, when compared to all other Ediacaran morphogroups, non-biomineral tubular organisms have the highest number of genera in both the White Sea assemblage and the Nama assemblage. The dominance of tubes in both assemblages shows that they made up a significant component of Ediacaran ecosystems for a considerably longer period of time than has previously been recognized. Notably, it is also found that the diversity of tubular organisms in the White Sea assemblage is broadly similar to the tubular fauna of the Nama assemblage, though there is a recorded increase in the compositional complexity of non-biomineral tubular taxa in the Nama assemblage.

## 2. Methods

The database developed for this study was constructed via data mining of peer-reviewed literature published before July 2023 and includes all occurrences of non-biomineral tubular organisms in the Ediacaran fossil record (electronic supplementary material, data set S1). The record of biomineral tubes is relatively well documented and well understood [4,13]. The aim of this study is to elucidate the non-biomineral tubular record; thus, only the non-biomineral members of the tubular morphogroup were included in the database. Results from this study provide new data that can be compared with results from previous research on biomineral tubes. This comparison is carried out in this study in order to contextualize the non-biomineral tubular record within the total morphogroup.

The term non-biomineral is here used to describe any tubular organism that is interpreted as having been originally non-rigid organic, rigid organic (e.g. kerogen) or non-biomineral to weakly biomineral. The latter category is included in our analyses because there is persisting uncertainty about the presence of biomineralization in the tubes categorized as such. The exclusion of biomineral taxa removed the majority of Cloudinomorphs from our analysis. However, the non-biomineral Cloudinomorphs (i.e. *Costatubus* spp., *Conotubus hemiannulatus*, *Convolotubus dargazinensis*, *Shaanxilithes ningqiangensis*, *Zuunia chimidtsereni*) were included because their unit-in-unit structure provides an interesting contrast to all other tubular organisms [4].

Here, a tubular organism is defined as any metazoan-grade organism with a single hollow and elongate form. Genera included in the database were formally described in the literature as tubular organisms. Any form that was described as ‘tubular’ but for which a macroalgal or single-cellular interpretation is preferred was excluded from the analysis (e.g. *Liulingjitaenia* [6], *Platysolenites* [31,32]). We also excluded forms comprised of multiple tubes (e.g. *Ernietta*) or multiple parts including a tube (e.g. *Namacalathus*) [33,34], because a single tube comprising the entirety of an organism is a distinct and commonly occurring form. Additionally, the definition of a tubular organism used here excludes the taxon *Plexus ricei* [35], which has in the past been described as a tubular organism but was re-evaluated by the authors subsequent to the compilation of the database, through which it became evident that *Plexus* is not a true tubular organism as its central axis never fully collapses, indicating that it was not hollow and thus does not fall within our definition of a tube. Additionally, the taxon *Thectardis avalonensis* of the Avalon assemblage was excluded from the analysis. Despite the fact that *Thectardis* possesses an overall morphology that is consistent with tubular organisms, it is not described as tubular in the literature, and it is most commonly grouped with Poriferan taxa, not tubes. While the authors would make the argument that *Thectardis* should be considered a tube based on outstanding definitions of the morphogroup, it was not included.

Individual entries in the database constructed for this study represent the occurrence of a non-biomineral tubular genus in a distinct fossil site or lithological unit (e.g. member or formation). Each entry does not indicate environmental, lithological, taphonomic or palaeogeographic distinction. Distinct fossil sites are defined as outcrops where tubular fossils occur that have different names and different modern geographic coordinates. These are used to characterize the occurence of non-biomineral tubes and do not imply palaeogeographic distinction.

The occurrence of non-biomineral tubes in palaeogeographically distinct localities was identified after data compilation based on the location of each fossil site within its broader locality and associated palaeocontinent, informed by reconstructions in Merdith *et al*. [36]. Disparate palaeogeographic locations were determined based on the distance and distribution of palaeocontinents, meaning that fossil sites that are estimated to have been associated with different palaeocontinents (e.g. Siberia and Baltica; North China and South China) are disparate. Fossil sites that are inferred to have been associated with the same palaeocontinents but are found in localities on distinct palaeoshorelines or are separated by a large distance are also considered to be disparate (e.g. the Wood Canyon Formation and the Deep Spring Formation are not disparate, but those Great Basin sites are considered to be disparate from the Miette Group of northwestern Canada despite all occurring on the palaeocontinent Laurentia). As such, many distinct occurrences in the database (i.e. individual entries) are found to occur in the same palaeogeographic location (electronic supplementary material, table S1; information supplied in the ‘Palaeocontinent’ column of electronic supplementary material, data set S1).

Entries in the database are resolved to the species level. However, only four genera are not monospecific (i.e. *Costatubus bibendi*, *C. kirsnovi*; *Saarina hagadorni*, *S. juliae*; *Sinospongia typica*, *S. chenjunyuani*; and *Vaveliksia velikanovi*, *V. svetozarovae*, *V. vana*). Some of these species concepts are based on the occurrence of distinct external characters, so the morphology-based component of this study is resolved to the species level. However, analysis of temporal patterns was conducted at the genus level because the majority of non-biomineral tubular taxa are monospecific, and this is a standard practice in broad-scale analysis of the Ediacaran biota. While this obfuscates differences between the species of non-biomineral tubular genera, these species concepts reflect differences in size and external features that do not impact the analyses or results pertaining to temporal patterns in non-biomineral tubes.

**Figure 1.**
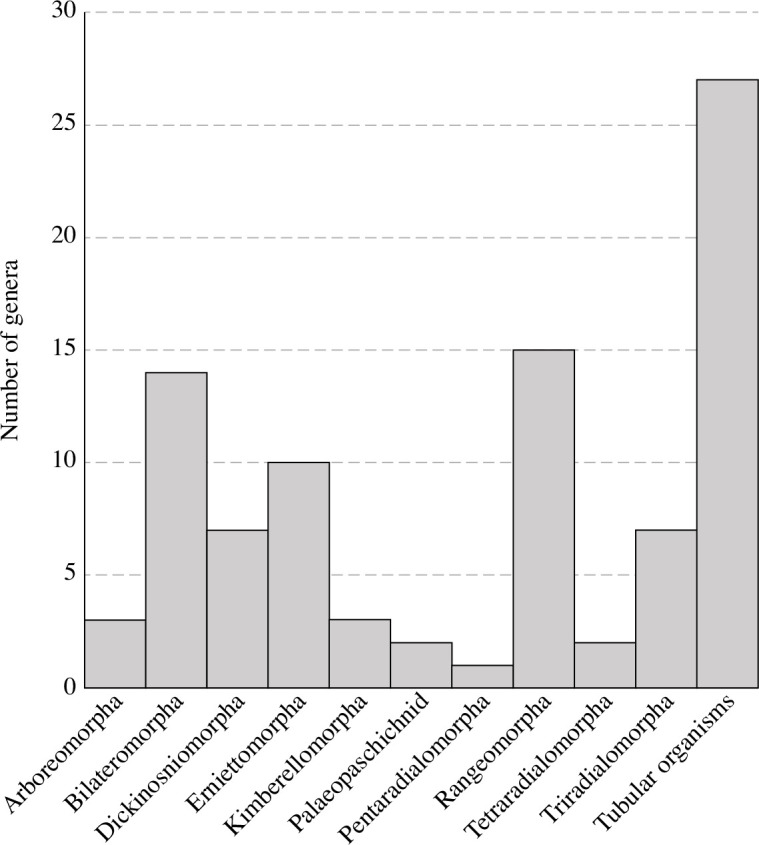
The number of genera in each major Ediacaran morphogroup, including genera present in the Avalon, White Sea and Nama assemblages.

Temporal ranges of non-biomineral tubular genera were ascertained through the compilation of the oldest and youngest occurrences of each genus. In many cases, non-biomineral tubes are preserved in formations that have poor geochronological constraints. This uncertainty was addressed by plotting occurrences within the assemblage only, resulting in an assemblage presence or absence assessment, not a range chart. While many of these organisms are thought to have had shorter temporal ranges, any other illustration of the data would be ignoring occurrences of tubular genera in poorly dated strata and/or presenting inaccurate ranges. For example, *Shaanxilithes* has a short stratigraphic range in China and Namibia, where dates are well constrained, but occurrences in other localities such as the Eastern European Platform, where some strata are less well constrained, call that short range into question. To compare the number of genera that comprise the non-biomineral subset of the tubular morphogroup and how this diversity changed through time with other Ediacaran morphogroups (e.g. figure 1; electronic supplementary material, table S2), the database compiled by Evans *et al*. [16] was used.

## 3. Results and discussion

### 3.1. Diversity

A primary goal of this study was to identify the number of non-biomineral tubular genera present in the Ediacaran. The database compiled for this study contains 108 non-biomineral tubular fossil occurrences, representing 27 genera distributed across 14 palaeogeographically distinct locations and 36 formations of the White Sea and Nama age (electronic supplementary material, table S1, data set S1). The broad palaeogeographic distribution of non-biomineral tubular organisms is consistent with previously documented patterns in the entirety of the Ediacaran biota and in subsets of tubular taxa [4,5,16]. However, the total number of non-biomineral tubular genera is higher than previously appreciated and is remarkably high when compared to the other non-tubular Ediacaran morphogroups (figure 1; electronic supplementary material, table S2). The non-tubular morphogroups with the highest number of genera are the Rangeomorpha, Bilateriomorpha and Erniettomorpha with 15, 14 and 10 genera, respectively. At 27 genera, non-biomineral tubes have a 45% higher number of genera than the most diverse non-tubular morphogroup, the Rangeomorpha. This previously unrecognized diversity of non-biomineral tubes demonstrates that a hollow, elongate form was the most common solution to complex life in the Ediacaran and, in conjunction with their high abundance in Ediacaran communities, suggests that this commonly occurring form was advantageous for life in Ediacaran oceans prior to the advent of biomineralization in Cloudinomorphs.

### 3.2. Morphological disparity

In addition to the identification of the number of non-biomineral tubular genera, this study aimed to describe the extent of morphological variability across non-biomineral tubes, including the assessment of gross morphological variability and diversity of external characters. While the idea that non-biomineral tubes vary morphologically is broadly understood, the extent and nature of this variability have not been characterized. The synthesis of the database reveals a high morphological disparity within the non-biomineral members of the tubular morphogroup despite their shared hollow and elongated forms. Overall morphology can be cylindrical, conical, ovular or comprised of nested growth units (i.e. Cloudinomorphs). This demonstrates that a cylindrical form is not diagnostic of all non-biomineral tubular taxa and that there is a suite of morphological features that can be used to place non-biomineral tubular taxa within a descriptive framework to provide a more robust definition of the total morphogroup.

**Figure 2.**
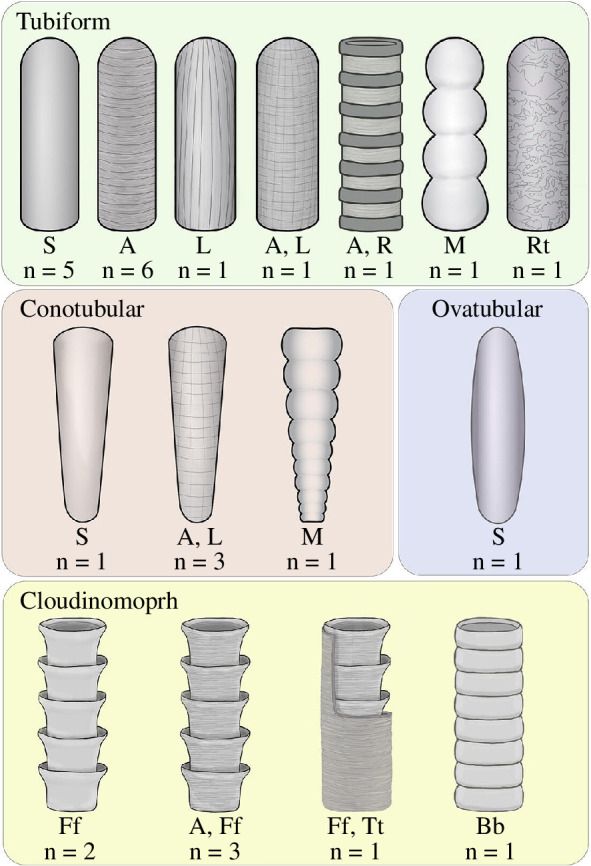
Illustration of the newly proposed form subgroupings and their external characters. *N* values are the number of genera within each subgrouping that possess the illustrated external features. External feature abbreviations: S, smooth; A, annulae; L, longitudinal striations; R, rings; M, modules; Rt, reticulation; Ff, funnel-in-funnel growth units; Bb, barrel-in-barrel growth units; Tt, tube in tube.

Cloudinomorphs, defined as biomineral and non-biomineral tubes constructed of nested and repeating collared cylindrical units, have previously been characterized as a subgrouping of the tubular morphogroup based on gross morphology and growth units [4]. The remaining non-biomineral tubes have not been similarly classified. To place all tubular taxa within a consistent classification framework that aids in the holistic description of the morphological variation in tubes, new subgroupings for tubular organisms, defined in a manner consistent with the previously established Cloudinomorph grouping, are proposed (figure 2). The proposed subgroupings include tubiform taxa, which have a cylindrical gross morphology, ovatubular taxa, which have an oval gross morphology that tapers at both ends, and conotubular taxa, which have a conical gross morphology that tapers basally. These groups were defined based on inferred dominant growth pattern, reflective of the fact that tubiform, conotubular and ovatubular morphologies are necessarily distinct in their dominant growth patterns. Because external characters are not robust indicators of growth patterns in the absence of detailed specimen-based analysis, they were not used to define the subgroupings. These subgroupings do not imply phylogenetic similarity or convergence and are intended to serve solely as an illustrative framework for defining the morphogroup with less generalization. Notably, external features such as annulae and modules do not appear to be characteristic of any one subgrouping. Instead, we observe these features occurring in multiple subgroupings (figure 2).

It is important to note that these new subgroupings are inclusive of all non-biomineral tubes but perpetuate the central problem with the tubular morphogroup in that it encompasses a prohibitively broad range of biological traits that, even with form-based subgroupings, are not phylogenetically meaningful. Thus, a classification scheme dependent solely on gross and constructional morphology is insufficient for identifying true synapomorphies in the tubular morphogroup. Furthermore, characterization of these subgroupings based on external characteristics (e.g. modules, annulae) can be misleading owing to convergence. Additionally, neither gross morphology nor external features serve as an accurate proxy for ecological strategies. Therefore, the extent of convergence, the phylogenetic affinities and the ecosystem impacts of tubular organisms as a total group remain unresolved and untestable.

Moving forward, the most effective strategy for defining subgroupings of tubular organisms should not aim to define phylogenetic similarity but instead be centred on establishing groups based on ecological similarity. This approach allows for the interpretation of the ecosystem impacts of tubular organisms without making untestable assumptions on phylogenetic similarity and has the potential to provide novel insight into the biological traits of tubular organisms as well. To aid in this endeavour, we suggest classifying constructional morphology into two groups: tubicolous construction and fluid-filled tubular construction. The former represents an organism living within an external tube with an open aperture and the latter represents an organism consists of a single body wall encompassing a fluid-filled interior with a closed aperture. These are distinct constructional morphologies that could also be convergently evolved but are certainly more biologically and ecologically meaningful than other methods of grouping. However, the data collected here reveal a dearth of tubular taxa that are definitively demonstrated to have had a closed or open aperture, and preservation of internal soft tissue is exceedingly rare [12].

As such, the existing understanding of tubular genera precludes the sorting of all tubular taxa into fluid-filled tubular forms and tubicolous tubular forms. That being said, the difference between Cloudinomorphs and all other tubular organisms is the best constraint we currently have on distinguishing between fluid-filled tubular organisms and tubicolous tubular organisms, as the characteristic multi-layered, nested, repeating growth units of Cloudinomorphs are most parsimoniously reconstructed as an external tube housing an organism. However, this is not a catch-all definition because several non-Cloudinomorphs are hypothesized to have had open apertures and internally dwelling organisms (e.g. *Calyptrina striata*) [15]. While the fossil evidence to corroborate these hypotheses is lacking, for example, the documentation of an open aperture is tenuous and there are no internal features preserved, it does cast doubt on the ability of the Cloudinomorph grouping to fully encompass all tubicolous forms.

This calls for a reassessment of known tubular genera with careful consideration of aperture morphology to instil more meaningful subcategories of tubular organisms that can be used to test broader hypotheses pertaining to the role of tubular organisms in terminal Ediacaran ecosystems.

### 3.3. Temporal variability

**Figure 3.**
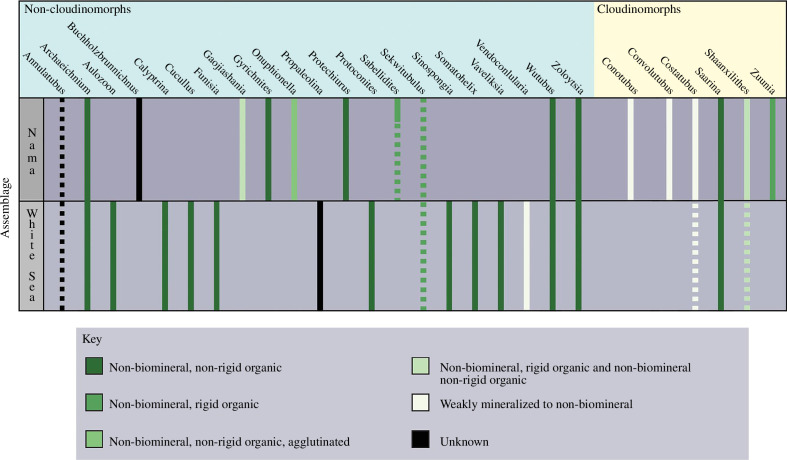
Schematic illustrating the presence or absence of non-biomineral tubular genera in the White Sea assemblage and the Nama assemblage. Dotted lines indicate genera with poor age constraints that potentially occur within the assemblage across which the line is dotted but cannot be definitively placed. Partially dotted lines (e.g. *Shaanxilithes*) represent genera for which some occurrences are well dated (solid lines), but other occurrences are poorly dated and could represent occurrences in the White Sea assemblage or the Nama assemblage (dotted lines). *Sabellidites* are well known to occur only in the terminal Nama assemblage, but there are several occurrences with poor age constraints. The line was dotted throughout the Nama assemblage to reflect this uncertainty but is not extended into the White Sea assemblage given their known restriction to the Nama assemblage. Occurrences represented solely by dotted lines are not considered in the analysis.

The second goal of this study was to describe the temporal variability in the number of genera and the constructional complexity of non-biomineral tubular organisms. This analysis is independent of the morphological sub-groupings defined above. When non-biomineral tubular genera are categorized as occurring in only the White Sea assemblage, only in the Nama assemblage, or in both the White Sea and the Nama assemblages, several newly recognized patterns in their diversity and constructional complexity (i.e. inferred original composition) through time are apparent (figure 3). Firstly, there are a minimum of 14 non-biomineral tubular genera present in the White Sea assemblage and 15 genera present in the Nama assemblage, with four of the genera from the White Sea assemblage carrying over into the Nama assemblage. Thus, the remarkably high number of non-biomineral tubular genera relative to other morphogroups is upheld within each assemblage. This demonstrates that the total morphogroup originated in and dominated the White Sea assemblage. Therefore, the dominance of the tubular morphogroup is not a signal driven by the radiation of non-biomineral tubes in the Nama assemblage, despite the appearance of novel non-biomineral tubular genera. Instead, the reign of the tubular morphogroup is firmly rooted within the White Sea assemblage, illustrating that a tubular morphology was not a unique solution to the environmental or ecological conditions of the Nama assemblage but was a long-ranging solution to complex multicellularity in Ediacaran oceans.

Notably, only four genera definitively occur in both assemblages. This constitutes a loss of 71% of tubular genera as a result of the White Sea–Nama extinction event [16]. This is marginally lower than the ~80% loss of genera calculated by Evans *et al*. [16] for all Ediacaran taxa. Non-biomineral tubes additionally record a remarkable recovery after the extinction event, regaining a similar number of genera subsequent to the event. This indicates that the extinction did not have a long-lasting effect on the ability of non-biomineral tubular organisms to originate and to dominate Ediacaran ecosystems.

The absence of a large increase in the number of non-biomineral tubular genera in the Nama assemblage calls into question the paradigm of the Nama assemblage as the age of tubes. When non-biomineral tubes are viewed in isolation, they indeed have a comparable number of genera in the White Sea and the Nama assemblages. However, the relatively depauperate Nama assemblage can appear to be uniquely dominated by the tubular morphogroup when relative diversity instead of absolute diversity is considered. The absolute diversity of non-biomineral tubes reported here demonstrates that they are not significantly more diverse in the Nama assemblage than in the White Sea assemblage; instead, non-biomineral tubes lost fewer taxa than the non-tubular Ediacaran morphogroups as a result of the White Sea–Nama extinction event and were more successful in their recovery. When viewed in the context of the total morphogroup, the dominance of tubes in the Nama assemblage does not represent an unprecedented increase in the number of non-biomineral tubular genera but is instead reflective of the radiation of biomineral tubular genera [4,13], the recovery of non-biomineral tubular genera, and a large decrease in the diversity of non-tubular Ediacaran morphogroups. Thus, the Nama assemblage may well have been the age of biomineral tubes but records no significant change in the dominance of non-biomineral tubes.

While the similarities between the non-biomineral tubes of the White Sea and the Nama assemblages suggest that the non-biomineral tubes were not broadly different between the two assemblages, there is a notably wider range of inferred original tissue compositions in Nama tubes than in White Sea tubes (figure 3). When genera with unknown original compositions are excluded, 86% of non-biomineral tubular genera in the White Sea assemblage were originally comprised of non-rigid organic material and 14% had weakly to non-biomineral original compositions. In contrast, 43% of non-biomineral tubular genera in the Nama assemblage were originally comprised of non-rigid organic material. The remaining 57% of non-biomineral tubes in the Nama assemblage had either all or some of their body wall comprised of rigid organic material, were agglutinated or were potentially very weakly biomineral to non-biomineral. This is in part driven by the radiation of non-biomineral Cloudinomorphs in the Nama assemblage, but this trend is also reflected in the inferred original compositions of the non-Cloudinomorph tubes (figure 3). This demonstrates that concomitant with the radiation of biomineral and non-biomineral Cloudinomorphs in the Nama assemblage [4,13,17], the non-Cloudinomorph tubular taxa were also becoming more complex and diverse in their biological traits.

In addition to changes in inferred original composition, the relative number of non-biomineral Cloudinomorph and non-Cloudinomorph tubular genera also varies between the White Sea and Nama assemblages. In the White Sea assemblage, non-biomineral Cloudinomorphs make up 13% of the total non-biomineral tubular diversity, which rises to 40% in the Nama assemblage. Concurrent with the increase in Cloudinomorph representation in the Nama assemblage, there is a minor loss in the number of non-Cloudinomorph tubular genera, which go from representing 93% of non-biomineral tubular diversity in the White Sea assemblage to 60% in the Nama assemblage. Despite this, non-Cloudinomorph taxa still make up the majority of non-biomineral tubular genera in the Nama assemblage. When taken together, the persisting dominance of non-Cloudinomorph tubular taxa and their increased variability in original composition in the Nama assemblage illustrate that the total tubular morphogroup, not just Cloudinomorphs, increased in complexity and played a large role in Ediacaran ecosystems during the Nama assemblage.

## 4. Conclusion

The compilation of the non-biomineral tubular record reveals several characteristics of the tubular morphotype that bolsters their status as key players in Ediacaran ecosystems, documents a previously unrecognized variability of gross morphology upon the tubular form and extends the range of their role as central components of Ediacaran ecosystems into the White Sea assemblage. This study demonstrates that non-biomineral tubes have the highest number of genera of all Ediacaran morphogroups, both within the Ediacaran biota as a whole and within the White Sea and the Nama assemblages. This is consistent with the understanding of tubes dominating the Nama assemblage, but importantly, it documents a previously unrecognized number of non-biomineral tubular genera in the White Sea assemblage. However, non-biomineral tubes of the Nama assemblage are found to have a higher variability in original compositions than tubes of the White Sea assemblage, indicating that the non-biomineral tubes followed similar patterns in increased complexity as did the biomineral Cloudinomorphs. The patterns revealed here show that, while all tubes increase in complexity in the Nama assemblage, they are characteristic and dominant components of the Ediacaran biota beginning in the White Sea assemblage.

It is additionally demonstrated that non-biomineral tubes were highly abundant for a longer period of time than the biomineral forms, leading to the conclusion that biomineralization was not required for the success (i.e. high diversity and abundance) of a tubular form. Thus, the tubular radiation of the Nama assemblage is a continuation of processes that began in the White Sea assemblage, with the perceived rapid increase in tubular forms in the Nama assemblage being a signal driven primarily by biomineral Cloudinomorphs. When viewed independently from the biomineral tubes, non-biomineral tubular organisms dominated the White Sea, persisted across the White Sea–Nama extinction event, and were re-established in the Nama assemblage. This long-ranging diversity of the non-biomineral tubular morphogroup is unmatched by any other non-tubular Ediacaran morphogroups, demonstrating that a tubular form was the most common solution to early multicellularity.

Further study of the total morphogroup as well as a detailed study of individual tubular genera is warranted to further elucidate the nature and implications of this long-ranging and widespread solution to complex life in Earth’s earliest animal ecosystems.

## Data Availability

The data used for this study are available as electronic supplementary material [37].
